# Fouling Release Coatings Based on Acrylate–MQ Silicone Copolymers Incorporated with Non-Reactive Phenylmethylsilicone Oil

**DOI:** 10.3390/polym13183156

**Published:** 2021-09-17

**Authors:** Hongwei Zhou, Yiming Zheng, Mengyu Li, Miao Ba, Yufeng Wang

**Affiliations:** Department of Material Science and Engineering, Changshu Institute of Technology, Changshu 215500, China; zhw12161999@163.com (H.Z.); a14533897772021@163.com (Y.Z.); LMENYU233@163.com (M.L.)

**Keywords:** fouling release, copolymer, phenylmethylsilicone oil, adhesion strength, leaching, antifouling performance

## Abstract

Copolymers containing MQ silicone and acrylate were synthesized by controlling the additive amount of compositions. Subsequently, fouling release coatings based on the copolymer with the incorporation of non-reactive phenylmethylsilicone oil were prepared. The surface properties of the coating (CAMQ_40_) were consistent with that of the polydimethylsiloxane (PDMS) elastomer, which ensured good hydrophobicity. Moreover, the seawater volume swelling rate of all prepared coatings was less than 5%, especially for CAMQ_40_ with only 1.37%. Copolymers enhanced the mechanical properties of the coatings, while the enhancement was proportional to the molar content of structural units from acrylate in the copolymer. More importantly, the adhesion performance between the prepared coatings and substrates indicated that pull-off strength values were more than 1.6 MPa, meaning a high adhesion strength. The phenylmethylsilicone oil leaching observation determined that the oil leaching efficiency increased with the increase in the structural unit’s molar content from MQ silicone in the copolymer, which was mainly owing to the decrease in compatibility between oil and the cured coating, as well as the decrease in mechanical properties. High oil leaching efficiency could make up for the decrease in the biofouling removal rate due to the enhancement of the elastic modulus. For CAMQ_40_, it had an excellent antifouling performance at 30 days of exposure time with more than 92% of biofouling removal rate, which was confirmed by biofilm adhesion assay.

## 1. Introduction

Biofouling of immersed artificial facilities is the accumulation and growth of unwanted biomolecules and organisms on surfaces through a dynamic process with considerable consequences [1,2], which is devastating for the ecological and economic status of the ocean industry [3,4]. Biofouling slows the speed of the ship hull with increasing fuel consumption and greenhouse gas emissions [5]. Other adverse effects include the spread of invasive species, damage to aquaculture, induce corrosion of metal structures, as well as threats to public health with toxic algae and bacteria [6,7,8].

Marine antifouling coatings applied by brushing can effectively inhibit marine fouling and has been recognized as the most widespread and cost-effective approach [9]. A better known example is the incorporation of tributyltin-based chemicals (TBT) as biocides in antifouling coatings, which have good antifouling effects with durability [5,10]. Unfortunately, further evaluation of chemical additives shows that the accumulation of severe toxicity has posed a serious threat to the marine ecological environment [9,11,12]. More serious, the threat has involved the survival of human beings [13,14]. Additionally, the chemical additive also includes a biocide based on copper compounds [7,11]. Owing to increased regulation, TBT was banned in 2008 by a convention set by the International Maritime Organization. Additionally, other biocides based on metal compounds are also facing increased scrutiny [15,16].

Although ongoing research on alternative non-metallic additives is still being processed, a very strong interest in developing entirely non-toxic coatings has led to the development trend of antifouling coatings [2,15,17,18,19]. Due to the complex and varied marine environment, as well as different attachment mechanisms by different fouling organisms, it is incredibly difficult to design a universal coating [4,9,20]. With the promulgation of regulations and legislations on prohibiting some toxic biocides, as well as limiting the use of other chemicals to decrease the influence on the environment [5,12], studies have been processed to increase the antifouling efficiency based on satisfying environmental protection requirements, which generally include (i) preventing biofouling from attaching to surface (fouling-resistant coatings), (ii) reducing biofouling adhesion strength on the immersed substrate surface (fouling-release coatings, FR coatings), and (iii) degrading/killing of biofoulings (fouling-degrading coatings) [2,5,6,14,21,22]. Additionally, the classification of the above eco-friendly antifouling coatings is not strictly limited. Various functional polymer antifouling coatings developed in recent years often have multiple antifouling functions [1,11,23,24].

FR coating is defined as one that cannot inhibit the attachment of fouling organisms, yet the interfacial bond between organisms and surface is severely weakened with low surface free energy (20–27 mJ/m^2^ of Baier curve) [6,23]. Besides, FR coatings usually have low elastic moduli [5]. Therefore, attached biofouling organisms can be readily removed by water shear force from ship hull navigation or mechanical cleaning. Typical FR coatings are based on silicone elastomers, especially polydimethylsiloxane (PDMS) elastomers [9,15,19]. Moreover, the silicone elastomer is also beneficial in terms of drag reduction via the smooth surface [25].

In the process of practical application, the final properties of silicone-based FR coatings are usually enhanced using additives to modify their mechanical resistance, adhesion strength to substrates, and fouling resistance to slime, which are the main disadvantages of silicone elastomers [14,26,27,28]. Currently, the incorporation of non-reactive silicone oil has aroused great interest in FR coating [28,29,30,31,32,33,34], which has been proved to be a non-toxic additive for the marine environment. Non-reactive silicone oil finally leaches to the surface between the coating and seawater, which can not only effectively improve the antifouling performance, especially the inhibition of barnacles’ adhesion [32], but also act as the flexible protective layer on the surface, to ensure the long-term smoothness of the coatings [23]. Therefore, non-reactive silicone oil additive has a wide application prospect in the FR coatings based on silicone. However, the adverse results also show that the incorporation of non-reactive silicone oil will further weaken the adhesion strength between the coatings and substrates [30], thereby limiting its utilization in antifouling coatings.

In this work, new copolymers containing methylvinyl MQ silicone and acrylic monomers were synthesized. MQ silicone resin is a new kind of silicone polymer material with a three-dimensional (non-linear) structure whose molecule structures are based on an –Si-O- bond. It is generally believed that MQ silicone resin belongs to a compact spherical body with a double-layer structure, as well as high crosslinking density. Additionally, the spherical body core is bonded by –Si–O- segments, while the spherical body shell is bonded by R_3_SiO_1/2_ groups with relatively low crosslinking density [35]. This unique molecular structure endows the flexible reactivity of MQ silicone resin. In this study, methylvinyl MQ silicone resin with olefin groups in the shell endows the ability to react with unsaturated groups, ensuring the synthesis by free radical polymerization. Based on the copolymers, 2-hydroxyethyl methacrylate phosphate acting as the adhesion promoter and non-reactive phenylmethylsilicone oil acting as an antifouling additive were incorporated to prepare the FR coatings. The properties of prepared coatings were measured such as the surface properties, swelling stability in seawater, and the mechanical properties (including the adhesion strength to substrates). Furthermore, studies of the oil leaching observation and seawater biofilm adhesion assay were done to evaluate the antifouling performance of the prepared coating. The aim is to develop a new and innovative strategy to prepare a good antifouling performance of FR coating with high adhesion strength to substrates.

## 2. Materials and Methods

### 2.1. Materials

Methylvinyl MQ silicone resin (MVMQ) with a vinyl group content of 0.08 mol/100 g was obtained from Dayi Chemical Industry Co., Ltd. (Yantai, China), the mole ratio of M:Q is 0.8:1. Methyl methacrylate, butyl methacrylate, isopropanol, and 2,2′-azobis(2-methylpropionitrile) (AIBN) were purchased from Sinopharm Chemical Reagent Co., Ltd. (Shanghai, China). Phenylmethylsilicone oil (PSO) with a viscosity of 30 mPa·s was obtained from CINDA Chemical Co., Ltd. (Bengbu, China). Acetone, toluene, xylene, ethanol, and acetylacetonate were supplied by Yongda Chemical Reagent Company (Tianjin, China). The compound 2-hydroxyethyl methacrylate phosphate (HEMAP) was supplied by YINCHANG XINCAI Company (Shanghai, China). The above chemicals were used for synthesis and related coating preparation, which were applied as received without any further purification. In addition, the commercial silicone resin was used for preparing the control FR coating, including the following raw materials: 10,000 mPa·s of hydroxyl-terminated polydimethylsiloxane (PDMS) from Dayi Chemical Industry Co., Ltd., tetraethylorthosilicate (TEOS) from Kemiou Chemical Co., Ltd. (Tianjin, China), and bismuth neodecanoate (BiND) from Deyin Chemical Co., Ltd. (Shanghai, China).

### 2.2. Synthesis of Acrylate–MVMQ Silicone Copolymer (AMQ)

**Figure 1:**
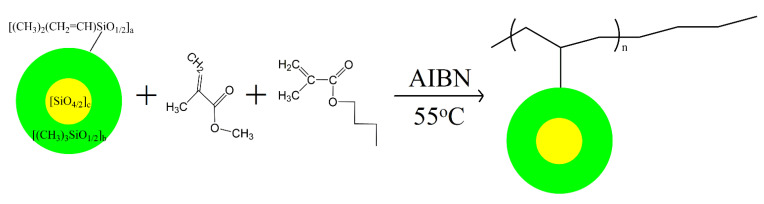
Synthesis of the AMQ.

In inception, methyl methacrylate, butyl methacrylate, AIBN, and mixed solvents of toluene, isopropanol, and acetone (3:1:6 of mass ratio) were added to a 500 mL four-necked round-bottom flask with a reflux device under an N_2_ atmosphere of which only 50% of the above chemicals were added at this stage. Then, MVMQ with all mass was added, and the reaction was stirred at 55 °C for 1 h. Subsequently, the remaining chemicals (methyl methacrylate, butyl methacrylate, AIBN, and mixed solvents) were injected into the reaction system in 0.5 h by using an LSP02-2B microinjection pump (Longer Pump Co., Ltd., Baoding, China). After 2.5 h of reaction, the reaction product was transferred into a triangular flask with a stopper and sealed at 25 °C for 24 h. Finally, the reaction product was washed at least thrice with ethanol to remove unreacted chemicals and solvents. After ethanol volatilization and filtration, the formed acrylate–MQ silicone copolymer (AMQ_x_) was obtained as a gel, and x represents the reactant composition mass of MVMQ. The synthesis of AMQ is shown in Figure 1. It should be noted that the gel remained sealed after drying. Additionally, the reactant composition of the synthesized AMQ copolymer was shown in Table 1.

### 2.3. Preparation of the FR Coating

By using a magnetic stirrer, AMQ and xylene with equal mass (100 g) were stirred in a sealed reagent bottle for 15 min at 200 rpm/min with 50 °C. Subsequently, PSO (5 g) and HEMAP (2 g) were added, respectively, and we continued to stir for 15 min. After that, it could be brushed. Teflon molds with dimensions of 150 × 150 × 5 mm^3^, tin plates with dimensions of 120 × 50 × 0.5 mm^3^, and glass culture dishes with a diameter of 60 mm and a thickness of 2 mm were applied as the substrates. The curing process was carried out in a vacuum at 25 °C for at least 8 h. Coatings cured in tin plates are to simulate brushing on the hull surface, which is mainly used for the analysis of surface properties, adhesion test, and antifouling performance. Teflon mold has very low surface free energy, so the cured films (5 mm of thickness) adhered to a Teflon mold can be easily removed. Additionally, the removed films can be cut for a swelling test and mechanical properties. Samples cured in glass culture dishes are used for the observation of leaching PSO. All measurements were carried out at room temperature. The experimental coating number is set as CAMQ_x_. In addition, the tin plates needed to be polished with 800 mesh sandpapers.

For the control coating (CS), the coating consisted of three parts, namely, the pre-dispersed (PDMS and PSO), curing agent (TEOS), and catalytic agent (BiND). A detailed description can be available elsewhere [30]. In particular, the additive amount of PSO is still 5 g in 100 g of PDMS. Additionally, the casting films and coatings were also cured with the Teflon molds, glass culture dishes, and tin plates.

In addition to the above preparation, the coating without incorporating PSO was also prepared separately. Additionally, films cast in Teflon molds were made, which were applied to the swelling test in sterilized seawater.

### 2.4. Analysis of the AMQ Copolymer

A spotlight 200I Fourier transfer infrared spectrophotometer (PerkinElmer Enterprise Management Co., Ltd., Shanghai, China) was used for the measurement of FT-IR spectra, which has a scan range of 4000–650 cm^−1^ and a resolution of 2 cm^−1^ by using the KBr disk method. For each sample, 32 scans were recorded. The surface layer and interface layer (interface layer between coating and substrate) FT-IR spectra of the prepared coatings were recorded using a high-performance diamond single-bounce attenuated total reflection accessory (ATR-IR), and the films cast in Teflon mold with 5 mm of thickness were used for the surface layer and interface layer ATR-IR spectra.

^1^H nuclear magnetic resonance (^1^H-NMR) spectroscopy was conducted on a Bruker AVANCE III HD (400 MHz, Bruker, Karlsruhe, Germany) in CDCl_3_ at 25 °C.

### 2.5. Surface Properties

Static contact angle (CA) including distilled water and diiodomethane was assessed with a JC2000C contact angle measurement system (Zhongchen Co., Shanghai, China). Three-microliter droplets of distilled H_2_O and CH_2_I_2_ were dripped on the sample surface using a syringe. Digital images of the droplet silhouette were captured with a charge-coupled device camera, and the CAs were calculated using the measuring angle method. Measurements were carried out at six points for each sample. Then, the surface free energy (SFE) value of the experimental sample was calculated by the Owens–Wendt two-liquid method [36].

The surface characteristics of the experimental coatings were analyzed by confocal laser scanning microscopy (CLSM) using an Olympus OLS4000 microscope (Olympus (China) Co., Ltd., Beijing, China). Additionally, the surface roughness (Sa) of samples was assessed by using the software of LEXT (version 2.2.4, Olympus (China) Co., Ltd., Beijing, China). All samples were cleaned with alcohol before the measurement.

### 2.6. Swelling Test

Prepared films ($m_{0}$) with a size of 20 × 20 × 5 mm^3^ were immersed in sterilized seawater. After t days, the films were taken out and rinsed in running water. Subsequently, the water on the film surface was soaked up with filter paper and weighed ($m_{t}$). The volume swelling rate of the film can be calculated by Formula (1)

(1) $$W_{s}=\frac{{(m}_{t}\;-\;m_{0})/\rho_{1}}{m_{0}/\rho }\;\times \;100\%$$

where W_s_ denotes the swelling rate in volume, and ρ represents the density of the prepared film. The density of seawater ($\rho_{1}$) was about 1 g/cm^3^.

### 2.7. Mechanical Properties

According to ISO 15184–1998, the pencil hardness of the coatings was evaluated by using a QH-A hand push pencil hardness tester (JXHG Co., Ltd., Tianjin, China) with a load of 750 g. The criterion for judging is that the number of the tested coating damages is no more than two in five tests of a pencil with a certain hardness mark. Additionally, the coating damage is defined as follows: the presence of a visible scratch or rupture in the surface of the films; material having been removed from the films.

According to GB/T 528-1998, dumbbell-shaped specimens were prepared for tensile strength measurement using a WDW-5 auto tensile tester (Xinbiao Automation Equipment Manufacturing Co., Ltd., Jinan, China) with 10 mm/min of a crosshead speed. Three samples of each coating were tested. The measure data with the strain less than 0.2 mm/mm were collected and fitted to obtain the elastic moduli of the coatings. Subsequently, the stress–strain curve of the coatings was determined by selecting a set of data in which the fitted elastic modulus was close to the average elastic modulus.

According to ISO 7619-1: 2004, the HT220 shore hardness A tester (Time High Technology Co., Ltd., Beijing, China) was used to evaluate the hardness of the coating. The coating thickness had to be over 2 mm for conducting the measurements, so the casting films with thickness above 4 mm were selected for the measurement.

### 2.8. Pull-Off Strength between the Coatings and Substrates

According to ISO 4624-2002, the pull-off strengths of the coatings were tested using a BGD500 portable adhesion tester with an accuracy of 0.01 MPa (Biuged Laboratory Instrument, Guangzhou, China). After the surface was cleaned with alcohol, an aluminum dolly with a diameter of 14 mm was adhered to the surface using epoxy adhesive (Araldite).

For the coating of CS, the standard measurement method needs to be improved due to the low SFE and low elastic modulus. Customized tin plate test dollies with a diameter of 14 mm were used for the test, and CS was brushed on the tin plate test dollies and the tin plate substrate at the same time. After 1 h of curing in vacuum at 25 °C, coated tin plate test dollies were bonded with the coated tinplate substrates. Then, the curing process needed to continue in a vacuum at 25 °C for at least 3 h. Next, the measurement was made. At least 6 samples of each coating would be tested.

### 2.9. Observation of Leaching PSO

PSO leached on the coating surface with exposure time was observed by a DYE-400E polarized light microscope with hot-stage (Dian Ying Optics, Shanghai, China) at 25 °C. At the early stage, the observation was carried out every 1 h to examine the exposure time required for leaching PSO (t_L_). Once PSO was observed in the field of view (1.231 × 0.924 mm^2^), the observation interval time could be determined for 1 day.

The mass of the glass culture dish was measured before the experiment. After 8 h of curing in a vacuum, the mass of the glass culture dish with film cast was measured. Then, the mass of the casting film (${\mathrm{Pm}}_{0}$) could be calculated. Subsequently, the mass of the casting film (${\mathrm{Pm}}_{t}$) was measured at t days after xylene dissolved the PSO on the surface. The leaching percent of PSO in the coating at t days ($R_{t}$) could be calculated with Formula (2), where m denotes the weight of PSO in the coatings. In addition, the exposure time required for leaching PSO was set as t_L_.

(2) $$R_{t}=\frac{{\mathrm{Pm}}_{0}{\;-\;\mathrm{Pm}}_{t}}{\mathrm{Pm}}\;\times \;100\%$$

### 2.10. Biofilm Adhesion Assay

The antifouling properties of the coating via the biofilm adhesion assay (marine bacteria) were evaluated about which a detailed description of the test was supplied elsewhere [23,30]. The brief description was as follows: six coated tin plate samples were immersed in 800 mL fresh seawater (China Yellow Sea) at 28 °C for 24 h, three samples were rinsed softly in sterile deionized water to remove unsettled bacteria, and the rest of the samples were washed by a CB-8L-C high-pressure water gun (Haishu Chebo Industry & Trade Co., Ltd., Ningbo, China) with 0.1 MPa for 120 s [23]; subsequently, samples were dyed with crystal violet solution (0.5 wt.%); after immersion in acetic acid solution (36 wt.%) for 10 min, the absorbance of the supernatant after centrifugation was measured at fixed wavelengths (590 nm) to evaluate the concentration of crystal violet via using an ultraviolet-visible spectrophotometer (NYSE: A, Palo Alto, CA, USA). The antifouling performance would be characterized by analyzing the absorbance of liquid, while the removal rate that marine bacteria adhered to the coating surface was also determined by Formula (3), where D_a_, D_b_, and R represent the absorbance of washed samples, the absorbance of rinsed samples, and the removal rate of biofilm adhesion.

(3) $$R=\frac{D_{b\;}{-\;D}_{a}}{D_{b}}\;\times \;100\%$$

For specimens of the biofilm adhesion assay, the entire surface of the tin plate had to be brushed by the prepared coating, to ensure that substrate corrosion owing to immersion in seawater did not affect the test results. Further, some coated tin plates were immersed in sterilized seawater for 30 days. We used a UV lamp for 15 min a day to ensure that no bacteria interfered with the specimens. After completing these operations, the coated tin plates were called the specimens after 30 days of exposure, while the coated tin plates used for testing just after 8 h of curing were called the specimens after 0 days of exposure.

## 3. Results and Discussion

### 3.1. Synthesis and Characterization of AMQ

New copolymers, named AMQ, were synthesized as designed to be applied to the silicone matrix to obtain FR marine coatings (Figure 1). Highly crosslinked MVMQ resin is considered to be a double-layer compact spherical structure, which is terminated by the trimethylsilyl groups. The trimethylsilyl groups ensure the excellent hydrophobicity of the MVMQ resin, while the vinyl groups can react with acrylic monomers by radical copolymerization. Additionally, two acrylic monomers selected were the commercial methyl methacrylate and butyl methacrylate. While AMQ was employed to prepare the FR coatings, the methyl group was conceived to provide the hydrophobic of the coating surface. At variance with the PDMS elastomer, AMQ synthesized by radical copolymerization can be well miscible with HEMAP through polar side chain, where HEMAP is often used as an adhesion promoter. After the prepared coating was cured, the well-adhesion performance on substrates was achieved by HEMAP, as well as the polar side chain of AMQ.

**Figure 2:**
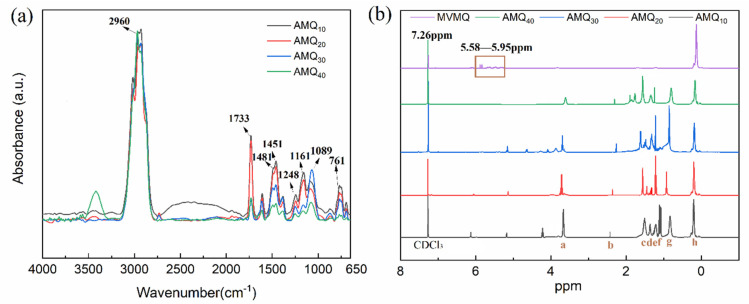
(**a**) The FT-IR spectra (KBr, r.t.); (**b**) ^1^H-NMR measurement of AMQ resin.

The structural characteristics of the AMQ with different reactant compositions were revealed by FT-IR spectroscopy (Figure 2a), and the detailed analysis was described as follows: 2960 cm^−1^ of the stretching vibration peak of –CH_3_, 1773 cm^−1^ of the featured absorption peak of –COOR, 1481 cm^−1^ and 1451 cm^−1^ of the bending vibration peak of -CH_2_-, 1248 cm^−1^ and 1161 cm^−1^ of the symmetric and anti-symmetric stretching vibration peaks of –C–O–C, 1089 cm^−1^ of the stretching vibration peak of –Si–O–Si-, and 761 cm^−1^ of the deformation vibration peak of –CH_3_. Compared with MVMQ (Supplementary File S1), the characteristic absorption peaks of the ester groups in the synthesized copolymer appeared at 1733 cm^−1^, 1248 cm^−1^, and 1161 cm^−1^. Moreover, the characteristic absorption peak at 1161 cm^−1^ was stronger than that at 1248 cm^−1^, indicating that the copolymer was synthesized with the reaction of MVMQ and two acrylic monomers. Further, the FT-IR spectra of the copolymer also determined that there was no peak at 1590 cm^−1^, confirming the absence of the alkene groups. The comparative analysis of copolymers showed that the intensity of the methyl group at 2960 cm^−1^ was enhanced gradually with the decrease in methyl methacrylate content and the increase in MVMQ content (x value) in the reactants, while the intensity of the ester group at 1733 cm^−1^, 1248 cm^−1^, and 1160 cm^−1^ decreased gradually. These differences showed that the molar content of the structural units derived from MVMQ in AMQ increased gradually, while those derived from acrylic monomers in AMQ decreased.

The assignments of ^1^H-NMR resonances for the AMQ with different reactant compositions are also shown in Figure 2b (H_a_, 3.88 ppm of –COOCH_3_; H_b_, 2.35 ppm of –COCH_2_-; H_c_,1.69 ppm of –CH_2_-; H_d_, 1.60 ppm of –C–CH_2_-C; H_e_, 1.38 ppm of –COC–CH_3_; H_f_, 1.26 ppm of –CH–Si–; H_g_, 0.93 ppm of –C–CH_3_; H_h_, 0.18 ppm of –Si–CH_3_). For the AMQ synthesized in this experiment, the characteristic signals of the methyl protons derived from methyl methacrylate, such as that at 3.88 ppm of –CO–O–CH_3_, butyl protons derived from butyl methacrylate, such as that at 0.93 ppm of –C–CH_3_ and 1.69 ppm of -CH_2_-, were recorded. Meanwhile, the alkene protons derived from the reactants of MVMQ resin were not observed (5.58–5.95 ppm) in Figure 2b, which means that the alkene groups of AMQ resin are completely reacted. Results of analysis also proved that the designed copolymer was successfully synthesized. Moreover, molar contents of the structural units in AMQ copolymers could also be determined by ^1^H-NMR based on the integral areas of the signals at 0.18 and 3.88 ppm, which are the characteristic signals of methyl protons derived from MVMQ and methyl methacrylate. The results also indicated that the molar content of the structural units derived from MVMQ in AMQ increased with the increase in MVMQ content, while that derived from acrylic monomers decreased.

### 3.2. Surface Properties

**Figure 3:**
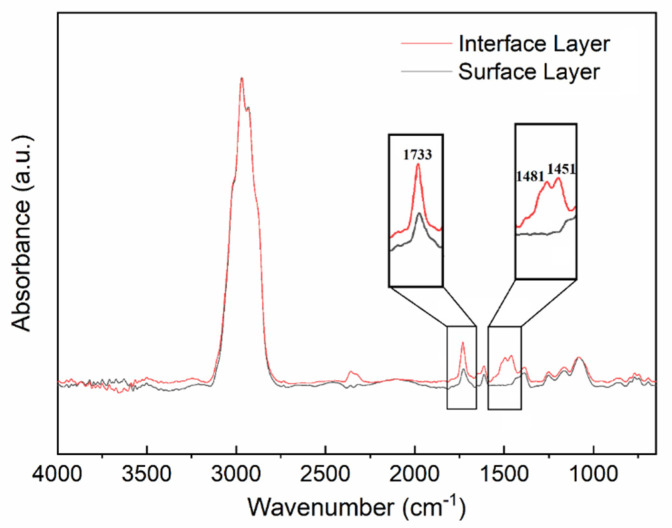
The FT-IR spectra of the coating (CAMQ40) surface and interface.

Static CA of the prepared coatings was measured using distilled water and diiodomethane as wetting liquids. The SFE was also calculated from static CA values by the Owens–Wendt two-liquid method. Table 2 lists static CAs and SFE values calculated. Compared with the CS, the water contact angle (WCA) of the prepared coating decreased. Yet, it was still more than 100°, maintaining good hydrophobicity. Although the newly AMQ copolymer containing the polar groups, as well as the incorporation of HEMAP, led to the enhancement of the polarity in the cured coatings, the molecular chain of AMQ caused migration and reconstruction during the curing of the prepared coating. It could be indicated by the FT-IR spectra of the coating surface layer and interface layer recorded using a high-performance diamond single-bounce attenuated total reflection accessory (Figure 3). By ATR-IR measurement, the infra-red from FT-IR mounted with ATR will penetrate the sample with micron-scale (or hundreds of nanometers), which is much less than 5 mm of the film thickness. Therefore, the ATR-IR analysis of the surface layer and interface layer can reflect the different molecular structures at different positions inside the prepared coatings, especially the migration of characteristic functional groups in the coatings. For the coating CAMQ_40_, the characteristic absorption peak intensity at 1733 cm^−1^ on the surface layer was lower than that on the interface layer. Moreover, no characteristic absorption peaks of the surface layer were recorded at 1481 cm^−1^ and 1451 cm^−1^. The above differences showed that the molar content of structural units derived from acrylic monomers in the cured copolymer at the coating surface layer is less than that at the coating interface layer. It also seemed that HEMAP was more concentrated in the interface layer of the coating. The migration of polar groups derived from the copolymer, as well as the enrichment of the polar additives on the interface layer, can form a strong hydrogen bond with the substrate, improving the adhesion strength, which can be analyzed by the pull-off test. For the hydrophobicity, the migration of polar groups ensured the non-polar performance of the coating surface, which was owing to the high concentration of the methyl groups (-Si-CH_3_) enriched on the prepared coating surface layer. As MVMQ content increased, the molar content of structural units derived from MVMQ in AMQ increased. Thus, the hydrophobicity of the prepared coating was further enhanced. Accordingly, the SFE value of the prepared coating decreased. For the prepared coating CAMQ_40_, the static WCA value was up to 107.8 ± 0.64°, which was close to that value of the CS. Additionally, the SFE value of CAMQ_40_ was 24.3 ± 0.67 mJ/m^2^, which satisfied the best SFE range for marine antifouling of the Baier curve [37,38].

**Figure 4:**
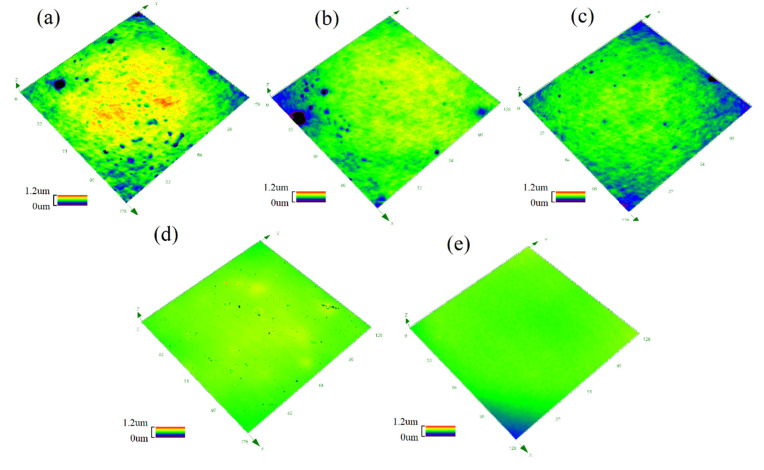
The surface morphology of the prepared coatings by the pseudo-color 3D mode of CLSM: (**a**) CAMQ_10_; (**b**) CAMQ_20_; (**c**) CAMQ_30_; (**d**) CAMQ_40_; (**e**) CS.

On the other hand, the hydrophobicity of the coatings was also related to the surface morphology observed in Figure 4 with the pseudo-color 3D mode by CLSM. Additionally, surface roughness data (Sa) are listed in Table 2. For the CAMQ_10_, the surface structure of the prepared coating was uneven due to the mutual repulsion of the polar groups and non-polar groups, resulting in the ups and downs of the morphology with a high Sa value measured. With the increase in MVMQ content, the molar content of the polar group in AMQ decreased. Additionally, the concentration of the non-polar group enriched on the surface increased (Figure 3). Thus, the uniformity of the coating surface structure was improved with more smoothness of the surface. Especially, the surface morphology of the CAMQ_40_ was close to that of the CS.

### 3.3. Swelling Stability in Seawater

**Figure 5:**
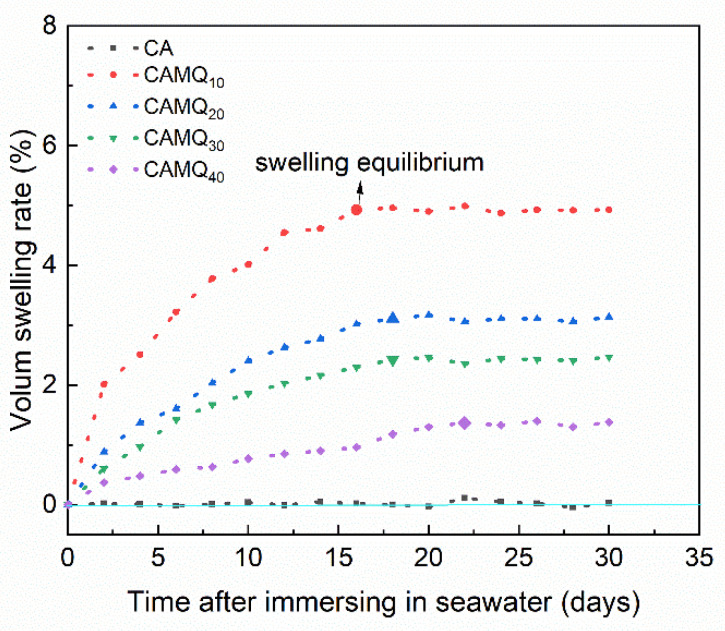
The volume swelling rate of the prepared coatings with the immersion time in seawater.

The volume swelling rate of the films immersed in seawater for 30 days was measured (Figure 5). For the CS, the cured PDMS elastomer had excellent seawater resistance, so its volume swelling rate was almost 0%. Whereas, the volume swelling rate of the prepared coatings decreased with the increase in MVMQ content with the maximum volume swelling rate below 5% (CAMQ10). Moreover, the volume swelling of all prepared coatings reached equilibrium in about 20 days and, then, made non-change. Methyl groups of the AMQ enriched on the coating surface had excellent hydrophobicity. It could resist the wetting of seawater, which also resisted the penetration of seawater into the prepared coatings. With the increase in MVMQ content, the molar content of structural units derived from MVMQ increased, meaning the increase in the concentration of methyl groups enriched on the coating surface. Additionally, the resisting seawater swelling property of the prepared coating was improved. For the film of CAMQ_40_, the volume swelling rate of the sample reached equilibrium in 22 days with only 1.37% of values, which showed excellent swelling stability in seawater. It was conducive to durability.

### 3.4. Mechanical Properties

**Figure 6:**
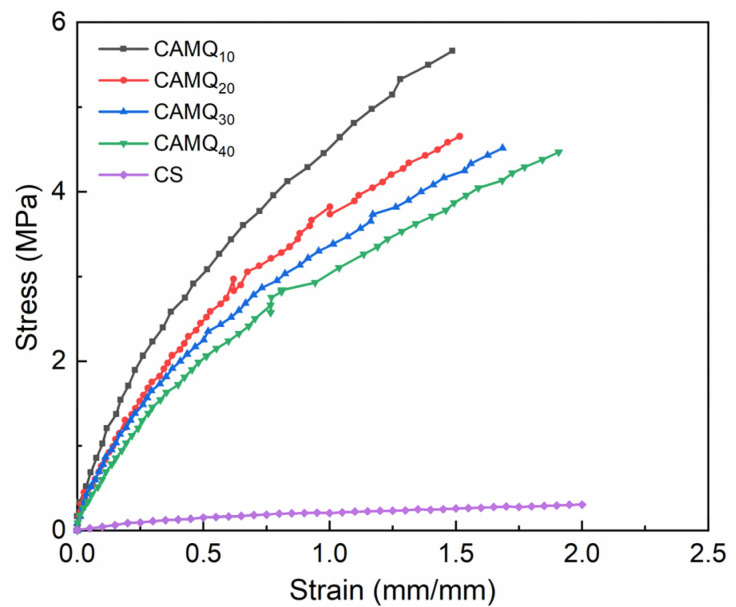
The stress–strain curve of the dumbbell-shaped samples from the cured film.

The commercial application of the FR coatings-based PDMS is limited due to the poor mechanical properties. Therefore, the PDMS elastomer must be reinforced and modified [6,11,14,23,39]. The stress–strain curves of the coatings are shown in Figure 6 by measuring the dumbbell-shaped samples from the cured film, and the related mechanical properties are listed in Table 3. The PDMS elastomer (the CS) had poor mechanical performance, and the related mechanical properties were far lower than that of the prepared coatings. For the prepared coatings based on AMQ, the mechanical properties decreased gradually with the increase in MVMQ content, yet it was still much higher than that of CS. As a hard monomer (T_g_ > 0 °C, and T_g_ represents glass transition temperature.), methyl methacrylate can enhance the hardness and adhesion strength of the copolymer, while MVMQ resin is a common strengthening and toughening material in the industry. Therefore, the prepared coatings based on AMQ copolymer had excellent mechanical properties. With the increase in the molar content of structural unit derived from MVMQ resin in AMQ copolymer, the mechanical properties (elastic modulus, tensile stress at 100%, and shore hardness) of the prepared coating decreased to a certain extent, yet the breaking elongation increased gradually, which was close to that of the CS. Pencil hardness is a measurement method used to evaluate the coating hardness. Compared with the CS, the pencil hardness of the prepared coatings was greatly enhanced, meaning that the prepared coatings could be better resistant to scratches in actual use. However, for the FR coating, the increase in elastic modulus would lead to the increase in energy consumption for the removal of marine fouling organisms adhered to the coating surface. It seemed to be detrimental to the marine antifouling performance of the coating. The difficulty of removing fouling organisms adhered to the coating surface depends on the shedding modes of fouling organisms [6,9]. When the elastic modulus of the coating is low, the shedding of fouling organisms tends to the peel-off mode. When the elastic modulus of the coating is high, it tends to the shear mode [12]. It also means that the work done when removing fouling organisms is equal to the deformation energy by the attachment of fouling organisms on the coating [5]. The low elastic modulus of the coating can ensure low deformation energy, which also ensures that the removal is less difficult. Accordingly, the Baier curve can also reflect the conclusion [37,38].

### 3.5. Pull-Off Strength Analysis

**Figure 7:**
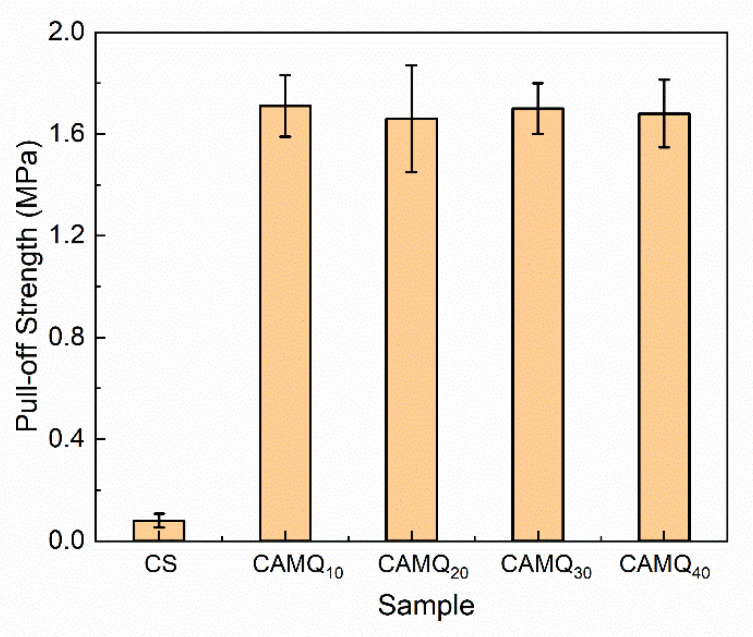
The pull-off strength value of the prepared coatings.

Improving the adhesion strength between the coatings and substrates is a challenge for the commercial application of the FR coatings-based PDMS. In this study, the pull-off strength evaluation was measured according to ISO 4624-2002 (Figure 7). The helically distributed methyl groups in the side chain of the PDMS resin ensure the excellent non-polarity of the cured PDMS elastomer, which would lead to the poor adhesion strength on the polarity substrates, such as tin plate, which has been indicated in the measured results of the CS. For the prepared coatings, the AMQ copolymers had both the non-polar methyl groups and the polar groups. During the curing process, the migration and reconstruction of the molecular chains ensured that the methyl groups migrated to the surface. Meanwhile, the polar groups migrated to the interface. More importantly, the HEMAP used as the adhesion promoter was incorporated in the prepared coating. It could improve the adhesion strength by forming a strong hydrogen bond with substrates. Relevant data analysis determined that the pull-off strength value of the prepared coating increased significantly, which was up to 1.7 MPa. Meanwhile, the pull-off strength value of the prepared coating did not change with the change in MVMQ content. It seemed that the incorporation of HEMAP played the main role in improving the adhesion strength between the prepared coatings and tin plates.

### 3.6. Leaching Behavior of PSO

**Figure 8:**
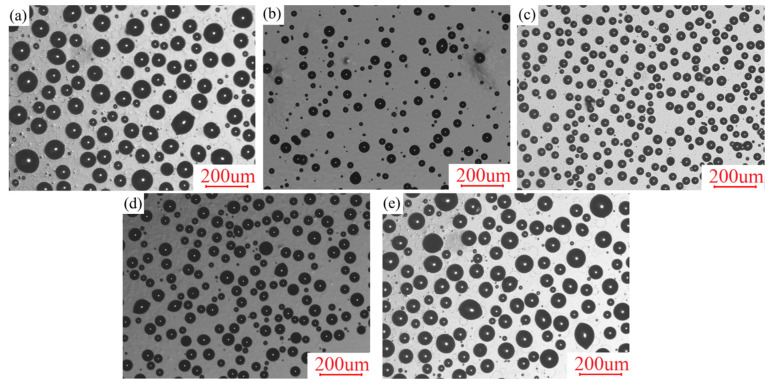
The surface morphology of the prepared with 30 days of exposure time (**a**) CS; (**b**) CAMQ_10_; (**c**) CAMQ_20_; (**d**) CAMQ_30_; (**e**) CAMQ_40_.

**Figure 9:**
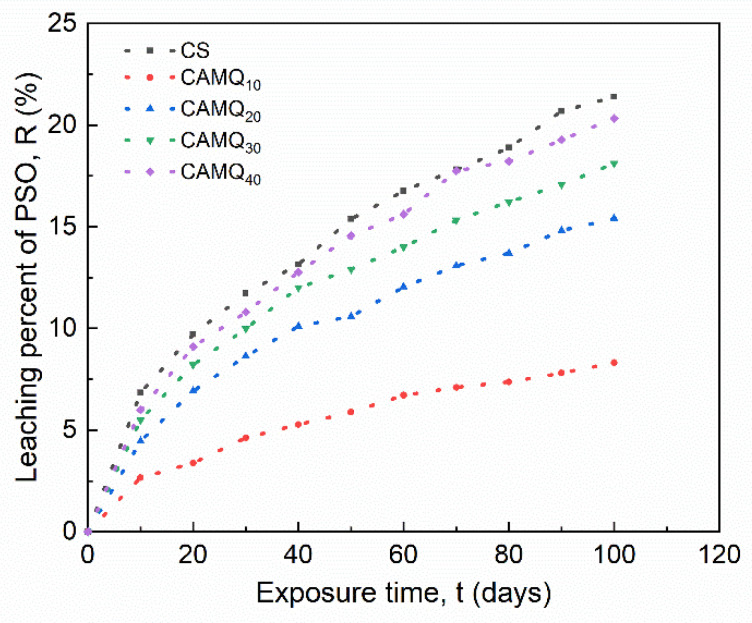
The leaching percent of PSO with exposure time.

By leaching PSO on the coating surface, the antifouling performance could be improved [29,32]. Thus, it is necessary to analyze the leaching behavior of the prepared coatings. After curing in a vacuum, PSO was gradually leached on the coating surface with the extension of exposure time. Figure 8 shows the surface morphology at 30 days of exposure time in ambient, while Table 4 indicates the related PSO leaching information. Additionally, Figure 9 shows the leaching percent of PSO with exposure time in ambient. It was obvious that PSO in the CS was easier to be leached. The poor mechanical properties of PDMS elastomer (CS) meant the low crosslinking density of the elastomer, which indicated a loose three-dimensional crosslinked structure [30,33,38]. Therefore, PSO could be efficiently leached to the coating surface (Figure 8a). For the prepared coatings, the leaching content of PSO on the coating surface increased at a fixed exposure time with the increase in MVMQ content. Especially for the CAMQ_40_, the leaching behavior and leaching efficiency ($t_{L}$ and $R_{100}$) of PSO were close to that of the CS coating (Table 4), which also meant that PSO could leach on the coating surface earlier, as well as more leaching content of PSO at the same exposure time. For the prepared coatings, the high mechanical properties ensured the excellent crosslinking density of the coating, so it could effectively inhibit the leaching of PSO. Different from better mutual solubility between PSO and PDMS elastomer, the driving force of PSO leaching would be generated by the polar groups of the synthetic copolymer (AMQ), as well as the incorporation of the HEMAP, which was due to the decrease in the compatibility between the cured coating and the PSO. With the decrease in the mechanical properties of the prepared coating, the leaching efficiency of PSO increased. Although the mechanical properties of the prepared coating were much better than those of CS coating, the PSO had poor compatibility with AMQ and HEMAP, which could promote the leaching of PSO. By the measurement and analysis of the PSO leaching behavior, it was determined that the prepared coatings of CAMQ_40_ had the ability of leaching PSO with good leaching efficiency.

### 3.7. Biofilm Adhesion Assay

**Figure 10:**
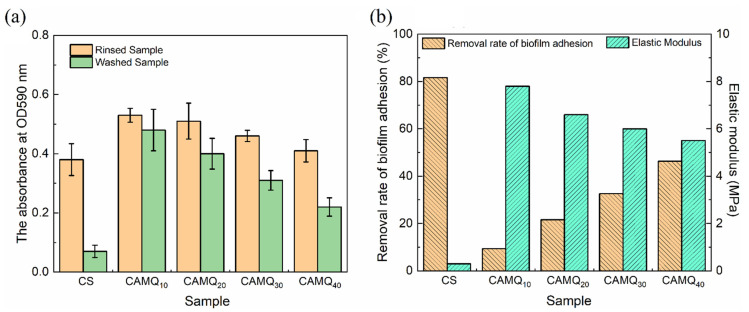
Biofilm adhesion results of the prepared coatings at 0 days of exposure time: (**a**) OD590 value; (**b**) fouling removal rate.

The antifouling performance was evaluated by the biofilm adhesion assay in this study, which was conducted with crystal violet staining by using a UV-vis spectrophotometer. The biofilm adhesion was tested at 0 days of exposure and 30 days of exposure, which could analyze the effect of leaching PSO on the antifouling performance. Figure 10a indicates the OD590 value of the rinsed samples and washed samples at 0 days, while Figure 10b is the results of the removal rates of the adhered biofilms. For the FR coatings, the adhesion of marine organisms is mainly determined by the surface free energy and elastic modulus [6,9,28,37]. Based on the characteristics of synthetic AMQ, the prepared coating had excellent hydrophobicity. Especially for CAMQ_40_, the SFE value was almost the same as that of the CS coating (Table 2). Yet, the elastic modulus of the prepared coatings was much larger than that of the CS (Table 3). With the increase in MVMQ content, the OD590 value of rinsed samples decreased. Low SFE could ensure that the coating surface was not easily adhered to by fouling organisms. Once the coating surface was adhered to by fouling organisms, the low elastic modulus performance could ensure that the adhering fouling organisms could be easily removed, which also resulted in the negative correlation between the removal rate and the elastic modulus. The removal rate of the adhered biofilms at 0 days of exposure reflected the same result. Although the fouling removal rate of the prepared coating increased with the increase in MVMQ content, the fouling removal rate value of the prepared coating was still larger than that of the CS coating. It proved that the high elastic modulus had a negative influence on the antifouling performance of the prepared coatings.

**Figure 11:**
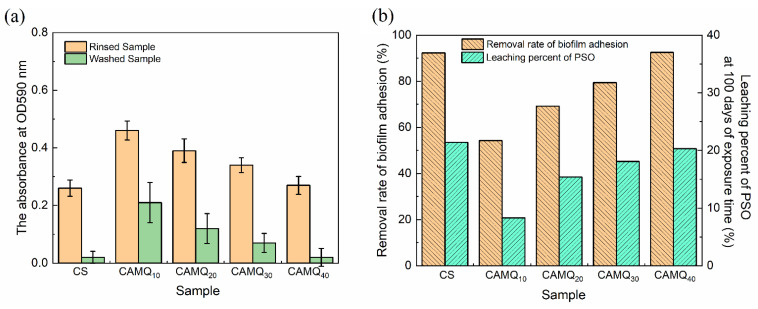
Biofilm adhesion results of the prepared coatings at 30 days of exposure time: (**a**) OD590 value; (**b**) fouling removal rate.

After 30 days of exposure time, PSO was leached on the coating surface. Additionally, the biofilm adhesion tests were conducted, focusing on the effect of leaching PSO on the removal rates of the adhered biofilms (Figure 11). With the increase in PSO leaching efficiency, the fouling removal rate of the prepared coating had been significantly improved. Especially for the prepared coating, CAMQ_40_, the fouling removal rate of the coating was close to that of CS. The PSO leached on the surface acted as a barrier between the coating surface and the fouling organisms. It ensured that fouling organisms were difficult to adhere to the coating surface. Even for the fouling organism adhered, it was easy to remove. Therefore, the antifouling performance of the coating with leaching PSO was mainly determined by the leaching efficiency of PSO. Results investigated that the biofilm adhesion removal rate of CAMQ_40_ was up to 92% at 30 days of exposure time, meaning excellent antifouling performance. It also indicated that the efficient leaching of PSO could effectively make up for the decrease in the antifouling performance due to the high elastic modulus.

## 4. Conclusions

New copolymers (AMQ) based on MVMQ resin were synthesized. Additionally, the characteristics of AMQ were analyzed by FTIR and ^1^H-NMR that the structural unit molar content of AMQ was changed according to the design with the change in MVMQ content. Subsequently, the FR coatings based on AMQ with PSO incorporated were prepared. During the curing process, the molecular chain of AMQ caused migration and reconstruction, which ensured the good hydrophobicity of the prepared coatings. With the increase in the structural unit molar content derived from MVMQ, the surface properties of the prepared coating were further improved, which included the hydrophobicity and surface smoothness. The volume swelling rate of all prepared coatings was less than 5%. With the increase in MVMQ content, the resisting seawater swelling property of the prepared coating was improved with only 1.37% of the volume swelling rate for CAMQ_40_. The prepared coatings had excellent mechanical properties. With the increase in the molar content of structural units derived from MVMQ resin in the copolymers, the mechanical properties (elastic modulus, tensile stress at 100%, and shore hardness) of the prepared coating decreased. Yet, the breaking elongation increased gradually, which was close to that of the CS. Further, the adhesion strength evaluation between the coatings and substrates indicated that the pull-off strength value of the prepared coatings was more than 1.6 MPa, comparing to the CS with only 0.08 MPa. It determined that the prepared coating showed excellent adhesion strength mainly due to the polar groups of the AMQ and HEMAP by forming a strong hydrogen bond with the substrates. Results of leaching PSO observation showed that the PSO leaching efficiency of the prepared coatings increased with the increase in MVMQ content, which was mainly dependent on the poor compatibility between PSO and the coatings, as well as the decrease in mechanical properties of the coatings. Although the high elastic modulus of the prepared coatings was not conducive to the antifouling performance, especially for the fouling removal property, the efficient leaching of PSO could make up for this deficiency. For the coating of CAMQ_40_, the adhered biofilm removal rate was more than 92% at 30 days of exposure time, exhibiting excellent anti-biofilm adhesion performance.

## Figures and Tables

**Table 1:** The reactant composition of the synthesized AMQ copolymer.

Chemicals and Reagents	AMQ_10_	AMQ_20_	AMQ_30_	AMQ_40_
Methyl methacrylate (g)	60	50	40	20
Butyl methacrylate (g)	30	30	30	30
MVMQ (g)	10	20	30	40
AIBN (g)	1	1	1	1
Mixed solvents (mL)	100	100	120	140

**Table 2:** The surface properties of the prepared coatings.

Sample	Static Contact Angles (°)		Surface Free Energy (mJ/m^2^)	Surface Roughness (Sa, μm)
	Water	Diiodomethane		
CAMQ_10_	100.7 ± 0.33	60.1 ± 0.70	28.6 ± 0.56	0.137
CAMQ_20_	103.8 ± 0.27	63.1 ± 0.63	26.9 ± 0.58	0.119
CAMQ_30_	105.5 ± 0.71	64.8 ± 0.54	26.0 ± 0.77	0.057
CAMQ_40_	107.8 ± 0.64	67.9 ± 0.38	24.3 ± 0.67	0.027
CS	109.8 ± 0.55	70.3 ± 0.52	22.9 ± 0.60	0.016

**Table 3:** The mechanical properties of the prepared coatings.

Scheme 100	Elastic Modulus (MPa)	Tensile Stress at 100% (MPa)	Breaking Elongation (%)	Shore Hardness (HA)	Pencil Hardness
CAMQ_10_	7.8 ± 1.01	4.45	148	60.7 ± 1.27	5H
CAMQ_20_	6.6 ± 0.77	3.82	151	54.5 ± 3.32	4H
CAMQ_30_	6.0 ± 0.69	3.38	168	48.8 ± 4.01	4H
CAMQ_40_	5.5 ± 1.22	3.05	191	41.1 ± 2.17	4H
CS	0.3 ± 0.04	0.21	200	12.5 ± 0.55	5B

**Table 4:** The exposure time required for leaching PSO and the leaching percent of PSO at 100 days of exposure time.

Sample	CS	CAMQ_10_	CAMQ_20_	CAMQ_30_	CAMQ_40_
$t_{L}$ (day)	0.4	3.1	1.6	1.1	0.8
$R_{100}$ (%)	21.4	8.3	15.4	18.1	20.3

## Data Availability

Data are contained within the article.

## Supplementary Materials

The following are available online at https://www.mdpi.com/article/10.3390/polym13183156/s1, File S1: the FTIR spectra of MVMQ.

## References

[1] Yang M.S., Sun Y.H., Chen G.M., Wang G.Y., Lin S.Z., Sun Z.Y. (2020). Preparation of a self-healing silicone coating for inhibiting adhesion of benthic diatoms. Mater. Lett..

[2] Maan A.M.C., Hofman A.H., de Vos W.M., Kamperman M. (2020). Recent Developments and Practical Feasibility of Polymer-Based Antifouling Coatings. Adv. Funct. Mater..

[3] Ware C.S., Smith-Palmer T., Peppou-Chapman S., Scarratt L.R.J., Humphries E.M., Balzer D., Neto C. (2018). Marine Antifouling Behavior of Lubricant-Infused Nanowrinkled Polymeric Surfaces. ACS Appl. Mater. Interfaces.

[4] Rasulev B., Jabeen F., Stafslien S., Chisholm B.J., Bahr J., Ossowski M., Boudjouk P. (2017). Polymer Coating Materials and Their Fouling Release Activity: A Cheminformatics Approach to Predict Properties. ACS Appl. Mater. Interfaces.

[5] Yang W.J., Neoh K.-G., Kang E.-T., Teo S.L.-M., Rittschof D. (2014). Polymer brush coatings for combating marine biofouling. Prog. Polym. Sci..

[6] Hu P., Xie Q.Y., Ma C.F., Zhang G.Z. (2020). Silicone-Based Fouling-Release Coatings for Marine Antifouling. Langmuir.

[7] Guazzelli E., Perondi F., Criscitiello F., Pretti C., Oliva M., Casu V., Maniero F., Gazzera L., Galli G., Martinelli E. (2020). New amphiphilic copolymers for PDMS-based nanocomposite films with long-term marine antifouling performance. J. Mater. Chem. B.

[8] Xie Q.Y., Pan J.S., Ma C.F., Zhang G.Z. (2019). Dynamic surface antifouling: Mechanism and systems. Soft Matter.

[9] Selim M.S., Shenashen M.A., El-Safty S.A., Higazy S.A., Selim M.M., Isago H., Elmarakbi A. (2017). Recent progress in marine foul-release polymeric nanocomposite coatings. Prog. Mater. Sci..

[10] Nurioglu A.G., Esteves A.C.C., de With G. (2015). Non-toxic, non-biocide-release antifouling coatings based on molecular structure design for marine applications. J. Mater. Chem. B.

[11] Azemar F., Fay F., Rehel K., Linossier I. (2020). Ecofriendly silicon-poly(lactic acid) hybrid antifouling coatings. Prog. Org. Coat..

[12] Eduok U., Faye O., Szpunar J. (2017). Recent developments and applications of protective silicone coatings: A review of PDMS functional materials. Prog. Org. Coat..

[13] Krishnan S., Weinman C.J., Ober C.K. (2008). Advances in polymers for anti-biofouling surfaces. J. Mater. Chem..

[14] Lejars M., Margaillan A., Bressy C. (2012). Fouling release coatings: A nontoxic alternative to biocidal antifouling coatings. Chem. Rev..

[15] Leonardi A.K., Ober C.K., Prausnitz J.M. (2019). Polymer-Based Marine Antifouling and Fouling Release Surfaces: Strategies for Synthesis and Modification. Annual Review of Chemical and Biomolecular Engineering.

[16] Zhang Z.-P., Song X.-F., Cui L.-Y., Qi Y.-H. (2018). Synthesis of Polydimethylsiloxane-Modified Polyurethane and the Structure and Properties of Its Antifouling Coatings. Coatings.

[17] Pinteus S., Lemos M.F.L., Alves C., Silva J., Pedrosa R. (2021). The marine invasive seaweeds Asparagopsis armata and Sargassum muticum as targets for greener antifouling solutions. Sci. Total. Environ..

[18] Leigh B.L., Cheng E., Xu L.J., Derk A., Hansen M.R., Guymon C.A. (2019). Antifouling photograftable zwitterionic coatings on PDMS substrates. Langmuir.

[19] Selim M.S., Elmarakbi A., Azzam A.M., Shenashen M.A., El-Saeed A.M., El-Safty S.A. (2018). Eco-friendly design of superhydrophobic nano-magnetite/silicone composites for marine foul-release paints. Prog. Org. Coat..

[20] Su C.M. (2017). Environmental implications and applications of engineered nanoscale magnetite and its hybrid nanocomposites: A review of recent literature. J. Hazard. Mater..

[21] Ren J., Han P., Wei H., Jia L. (2014). Fouling-resistant behavior of silver nanoparticle-modified surfaces against the bioadhesion of microalgae. ACS Appl. Mater. Interfaces.

[22] Banerjee I., Pangule R.C., Kane R.S. (2011). Antifouling coatings: Recent developments in the design of surfaces that prevent fouling by proteins, bacteria, and marine organisms. Adv. Mater..

[23] Fan F.-X., Zheng Y.-M., Ba M., Wang Y.-F., Kong J.-J., Liu J.-H., Wu Q. (2021). Long time super-hydrophobic fouling release coating with the incorporation of lubricant. Prog. Org. Coat..

[24] Shi S., Li B., Qian Y., Mei P., Wang N. (2020). A simple and universal strategy to construct robust and anti-biofouling amidoxime aerogels for enhanced uranium extraction from seawater. Chem. Eng. J..

[25] Galhenage T.P., Webster D.C., Moreira A.M.S., Burgett R.J., Stafslien S.J., Vanderwal L., Finlay J.A., Franco S.C., Clare A.S. (2017). Poly(ethylene) glycol-modified, amphiphilic, siloxane-polyurethane coatings and their performance as fouling-release surfaces. J. Coat. Technol. Res..

[26] Selim M.S., El-Safty S.A., El-Sockary M.A., Hashem A.I., Elenien O.M.A., El-Saeed A.M., Fatthallah N.A. (2016). Smart photo-induced silicone/TiO_2_ nanocomposites with dominant 110 exposed surfaces for self-cleaning foul-release coatings of ship hulls. Mater. Des..

[27] Liu C., Xie Q., Ma C., Zhang G. (2016). Fouling Release Property of Polydimethylsiloxane-Based Polyurea with Improved Adhesion to Substrate. Ind. Eng. Chem. Res..

[28] Galhenage T.P., Hoffman D., Silbert S.D., Stafslien S.J., Daniels J., Miljkovic T., Finlay J.A., Franco S.C., Clare A.S., Nedved B.T. (2016). Fouling-Release Performance of Silicone Oil-Modified Siloxane-Polyurethane Coatings. ACS Appl. Mater. Interfaces.

[29] Ba M., Zhang Z.-P., Qi Y.-H. (2018). The leaching behavior of phenylmethylsilicone oil and antifouling performance in nano-zinc oxide reinforced phenylmethylsilicone oil-Polydimethylsiloxane blend coating. Prog. Org. Coat..

[30] Ba M., Zhang Z., Qi Y. (2018). Fouling Release Coatings Based on Polydimethylsiloxane with the Incorporation of Phenylmethylsilicone Oil. Coatings.

[31] Selim M.S., Shenashen M.A., Elmarakbi A., El-Saeed A.M., Selim M.M., El-Safty S.A. (2017). Sunflower oil-based hyperbranched alkyd/spherical ZnO nanocomposite modeling for mechanical and anticorrosive applications. RSC Adv..

[32] Shivapooja P., Cao C., Orihuela B., Levering V., Zhao X., Rittschof D., Lopez G.P. (2016). Incorporation of silicone oil into elastomers enhances barnacle detachment by active surface strain. Biofouling.

[33] Fernández Estarlich F.M., Eaton P.J., Fletcher R.L., Lewey S.A., Nevell T.G., Smith J.R., Tsibouklis J. (2012). The Effects of Incorporated Silicone Oils and Calcium Carbonate on the Resistance to Settlement and the Antifouling Performance of a Silicone Elastomer. J. Adhes. Sci. Technol..

[34] Nendza M. (2007). Hazard assessment of silicone oils (polydimethylsiloxanes, PDMS) used in antifouling-/foul-release-products in the marine environment. Mar. Pollut. Bull..

[35] Liang W.J., Ge X., Ge J.F., Li T.H., Zhao T.K., Chen X.J., Song Y.Z., Cui Y.D., Khan M., Ji J.Y. (2018). Reduced Graphene Oxide Embedded with MQ Silicone Resin Nano-Aggregates for Silicone Rubber Composites with Enhanced Thermal Conductivity and Mechanical Performance. Polymers.

[36] Owens D.K., Wendt R.C. (1969). Estimation of the surface free energy of polymer. J. Appl. Polym. Sci..

[37] Baier R.E. (2006). Surface behaviour of biomaterials: The theta surface for biocompatibility. J. Mater. Sci. Mater. Med..

[38] Brady R.F., Singer I.L. (2000). Mechanical factors favoring release from fouling release coatings. Biofouling.

[39] Ba M., Zhang Z.P., Qi Y.H. (2019). The influence of MWCNTs-OH on the properties of the fouling release coatings based on polydimethylsiloxane with the incorporation of phenylmethylsilicone oil. Prog. Org. Coat..

