# Supplementary material for: Fouling Release Coatings Based on Acrylate–MQ Silicone Copolymers Incorporated with Non-Reactive Phenylmethylsilicone Oil
Source: Polymers (Basel). 2021 Sep 17;13(18):3156. doi: 10.3390/polym13183156 (PMC8469071; doi:10.3390/polym13183156)
Supplement: Supplementary file 1 [file polymers-13-03156-s001.zip › Supplementary File 1.pdf]

For the FT-IR spectra of AMQ

2960 cm^{-1}: the stretching vibration peak of $-CH_3$

1733 cm^{-1}: the featured absorption peak of -COOR. It indicates that the monomer has radical polymerization with VMQ resin.

1481 cm-1: the bending vibration peak of $–CH_2-$

1451 cm^{-1}: the bending vibration peak of $–CH_2-$. Because of the reaction between the monomer and VMQ, the peak exists in the newly synthesized resin.

1248 cm^{-1} and 1161 cm^{-1}: the symmetric and anti-symmetric stretching vibration peaks of –C-O-C- in ester group. Meanwhile, the peak at 1170 cm^{-1} is stronger, which means that the reaction product is the acrylic modified VMQ silicone resin, instead of poly-acrylate formed by radical polymerization of acrylic monomers.

1089 cm^{-1}: the stretching vibration peak of –Si-O-Si-

761 cm^{-1}: the deformation vibration peak of $–CH_3$
