# Supplementary material for: Fouling Release Coatings Based on Acrylate–MQ Silicone Copolymers Incorporated with Non-Reactive Phenylmethylsilicone Oil
Source: Polymers (Basel). 2021 Sep 17;13(18):3156. doi: 10.3390/polym13183156 (PMC8469071; doi:10.3390/polym13183156)
Supplement: Supplementary file 1 [file polymers-13-03156-s001.zip › Supplementary File 2.pdf]

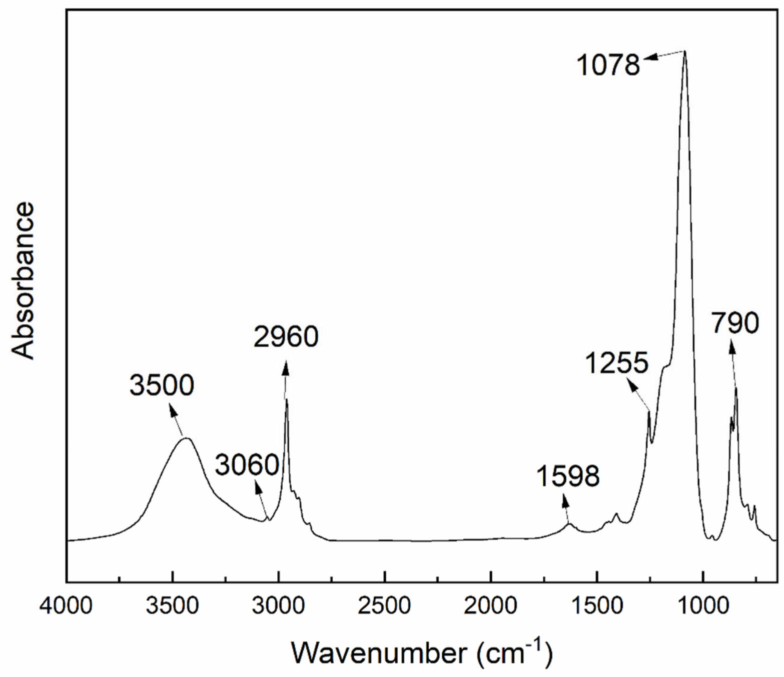

For the FT-IR spectra of MVMQ

3500 cm^{-1}: the featured absorption peak of –OH. Due to the production and synthesis process of VMQ resin, there must be hydroxyl groups.

3060 cm^{-1}: the stretching vibration peak of -C-H in CH_2=CH-

2960 cm^{-1}: the stretching vibration peak of CH_3-

1598 cm^{-1}: the stretching vibration peak of –C=C- in CH_2=CH-

1255 cm^{-1}: the stretching vibration peak of CH_3-Si-

1078 cm^{-1}: the stretching vibration peak of –Si-O-Si-

790 cm^{-1}: the stretching vibration peak of –$Si(CH_3)_2$. Due to exist symmetric and asymmetric peaks, there are multiple peaks in nearby wavenumber.
