# Supplementary material for: Fouling Release Coatings Based on Acrylate–MQ Silicone Copolymers Incorporated with Non-Reactive Phenylmethylsilicone Oil
Source: Polymers (Basel). 2021 Sep 17;13(18):3156. doi: 10.3390/polym13183156 (PMC8469071; doi:10.3390/polym13183156)
Supplement: Supplementary file 1 [file polymers-13-03156-s001.zip › Supplementary File 3.pdf]

For ^{1}H-NMR spectra of AMQ

0.05 ppm: -Si-CH_3

0.84 ppm: -C-CH_3

1.13 ppm: -CH-Si-

1.26 ppm: -CO-C-CH_3

1.47 ppm: -C-CH-C

1.57 ppm: -CH_2-

2.22 ppm: -CO-CH_2-

3.79 ppm: -CO-O-CH_3
